# Physiological Benefits of Oxygen-Terminating Extracellular Electron Transfer

**DOI:** 10.1128/mbio.01957-22

**Published:** 2022-11-14

**Authors:** Yoshihide Tokunou, Masanori Toyofuku, Nobuhiko Nomura

**Affiliations:** a Faculty of Life and Environmental Sciences, University of Tsukuba, Ibaraki, Japan; b International Center for Materials Nanoarchitectonics, National Institute for Materials Science, Ibaraki, Japan; c Microbiology Research Center for Sustainability, University of Tsukuba, Ibaraki, Japan; China University of Geoscience in Wuhan; Ohio State University

**Keywords:** extracellular electron transfer, oxygen reduction, energy metabolism, oxygen gradient, biofilms

## Abstract

Extracellular electron transfer (EET) is a process via which certain microorganisms, such as bacteria, exchange electrons with extracellular materials by creating an electrical link across their membranes. EET has been studied for the reactions on solid materials such as minerals and electrodes with implication in geobiology and biotechnology. EET-capable bacteria exhibit broad phylogenetic diversity, and some are found in environments with various types of electron acceptors/donors not limited to electrodes or minerals. Oxygen has also been shown to serve as the terminal electron acceptor for EET of Pseudomonas aeruginosa and Faecalibacterium prausnitzii. However, the physiological significance of such oxygen-terminating EETs, as well as the mechanisms underlying them, remain unclear. In order to understand the physiological advantage of oxygen-terminating EET and its link with energy metabolism, in this review, we compared oxygen-terminating EET with aerobic respiration, fermentation, and electrode-terminating EET. We also summarized benefits and limitations of oxygen-terminating EET in a biofilm setting, which indicate that EET capability enables bacteria to create a niche in the anoxic zone of aerobic biofilms, thereby remodeling bacterial metabolic activities in biofilms.

## INTRODUCTION

Extracellular electron transfer (EET) refers to a process that enables bacterial electrons to be exchanged across their membrane by donating electrons to extracellular materials or accepting electrons from them (1). Bacterial EET was first studied for the reactions on minerals and electrodes with implication in geobiology and biotechnology (2, 3). Solid Fe(III) oxide shows low solubility in water; thus, the dissimilatory microbial reduction of Fe(III) oxide into soluble Fe(II) via EET impacts Earth’s iron cycle (4, 5). Based on bacteria-induced reduction of solid Fe(III), EET-capable bacteria have been utilized for microbial electrochemical technologies, such as microbial fuel cells, where bacterial respiratory electrons are donated to extracellular electrodes generating electrical energy (3, 6). Subsequent studies have demonstrated that terminal electron acceptors/donors for EET process are not limited to minerals and electrodes and electrical link is formed with other cells (7, 8), humic substances (9), soluble extracellular shuttling molecules (10), etc. However, most EET studies have focused on reactions involving membrane impermeable redox molecules because EET reacts with extracellular molecules.

**FIG 1:**
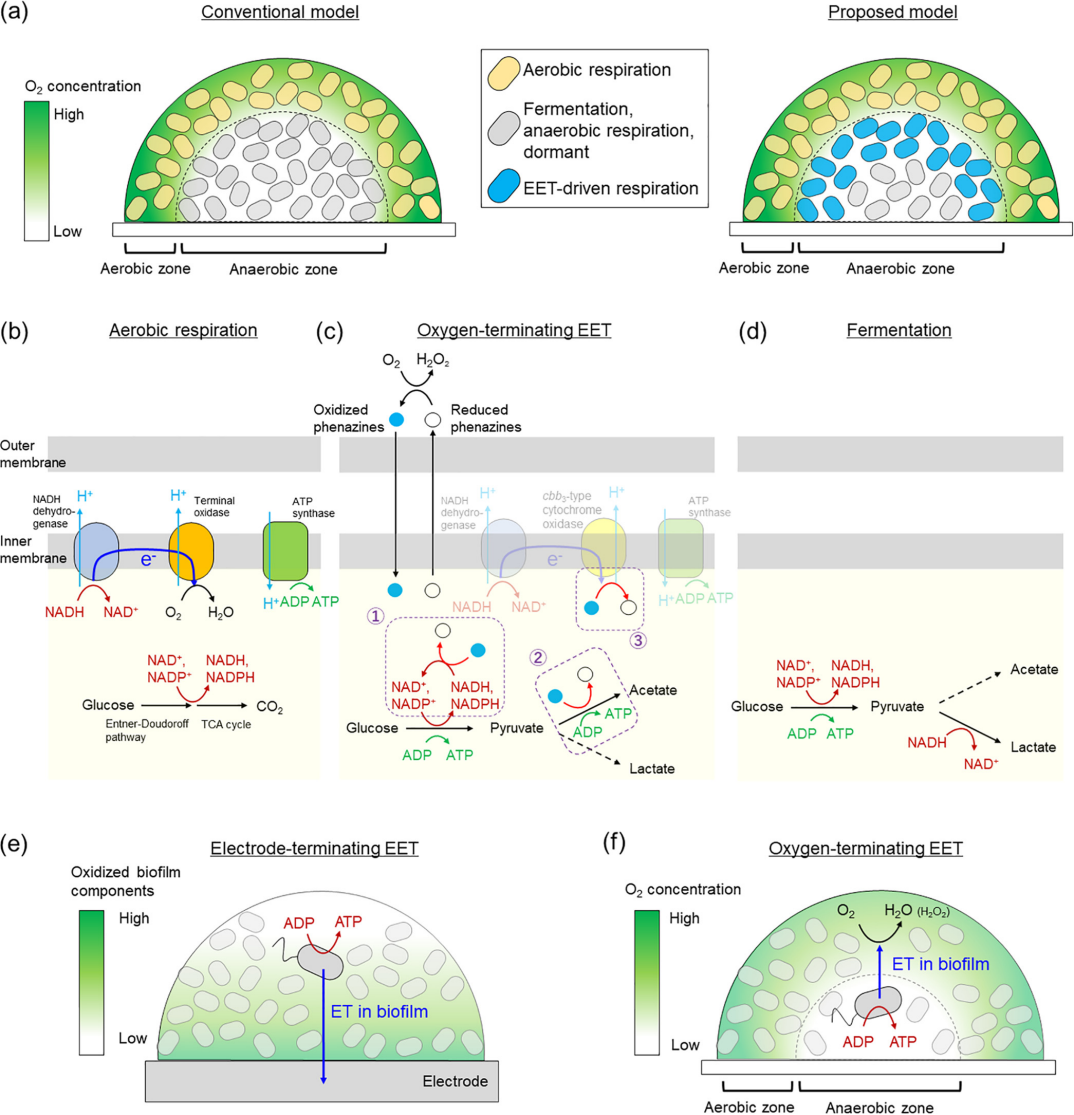
(a) Oxygen concentration gradient and bacterial subpopulations in biofilms. Energy conservation mechanisms of (b) aerobic respiration, (c) oxygen-terminating extracellular electron transfer (EET), and (d) fermentation using Pseudomonas aeruginosa as a model system. Phenazines are proposed to be reduced intracellularly by ① NADH or NADPH; ② some enzymes, including pyruvate dehydrogenase; and ③ *cbb*_3_-type cytochrome oxidase. Proton pumping capability coupled with phenazine reduction has not been demonstrated. (e) Electrode-terminating EET and (f) oxygen-terminating EET in biofilms where electron transfer (ET) in biofilms sustains bacterial energy conservation distant from electrode or oxygen.

Oxygen is permeable to bacterial membrane but acts as a terminal electron acceptor for EET (here we refer this to “oxygen-terminating EET”). Khan et al. demonstrated that Faecalibacterium prausnitzii, a Gram-positive anaerobe inhabiting human guts, can shuttle riboflavin and thiols to reduce extracellular oxygen which contribute to thrive in the interface of anoxic and oxic zones in the intestine (11). Similarly, Pseudomonas aeruginosa, an opportunistic Gram-negative bacterium that causes several infections (12), reduces extracellular oxygen using self-secreted phenazines (13). However, oxygen-terminating EET, which has been observed specifically in *F. prausnitzii* and P. aeruginosa, is often discussed separately from that of other versatile EET-capable bacteria terminating electrodes or minerals, indicating that the understanding of its mechanisms and physiological significance is improper. Here, we unify recent progress in oxygen-terminating EET related research by comparing their energy conservation mechanisms with those of aerobic respiration, fermentation, and electrode-terminating EET. We focus on oxygen-terminating EET in biofilms and discuss possible limitations affecting the sustenance of bacterial metabolism via such a process. These lead the proposal that one of the advantages associated with EET-capability is the creation of a niche in the presence of an oxygen gradient, thereby remodeling metabolic activities in biofilms (Fig. 1a).

## ENERGY CONSERVATION MECHANISMS OF OXYGEN-TERMINATING EET IN COMPARISON WITH OTHER RESPIRATION METHODS

To clarify the physiological benefit of oxygen-terminating EET and to understand the energy conservation mechanisms, we summarized recent progress in oxygen-terminating EET of P. aeruginosa and *F. prausnitzii*. As both *F. prausnitzii* and P. aeruginosa can conserve energy with other pathways, we compare the oxygen-terminating EET mechanisms with those of fermentation, aerobic respiration, and electrode-terminating EET, introducing molecular mechanisms of oxygen-terminating EET in P. aeruginosa.

### Comparison with aerobic respiration.

Oxygen-terminating EET has disadvantages in the efficiency to generate ATP due to the location occurring the oxygen reduction reaction. In aerobic respiration, oxygen reduction is catalyzed by inner membrane terminal oxidases, which pump protons across the membrane via respiratory electron transfer, generating a proton gradient. Although P. aeruginosa possesses five terminal oxidases with multiple isoforms exhibiting different proton-pumping activity levels that act under different conditions (14, 15), oxidative phosphorylation occurs via a proton motive force, producing 38 ATP from one glucose during typical bacterial aerobic respiration (Fig. 1b) (16). In contrast, when oxygen-terminating EET proceeds, protons are not pumped via oxygen reduction due to the absence of intracellular oxygen reduction reaction. Instead, P. aeruginosa use soluble electron shuttles, phenazines, which deliver electrons from intracellular NAD(P)H to extracellular oxygen, leading to ATP production (13). Two ATP are generated associated with oxygen-terminating EET via the Entner-Doudoroff pathway with subsequent pyruvate fermentation to acetate when glucose is provided as a carbon source to P. aeruginosa (Fig. 1c) (17). Oxidized phenazines directly accept electrons from NAD(P)H during ATP production on glucose (18), which regenerate NAD(P)^+^ for further ATP production. Reduced phenazines are subsequently oxidized by extracellular oxygen, enabling sustainable production of NAD(P)^+^ and ATP (Fig. 1c). An enzymatic assay using cell lysates of P. aeruginosa indicated that the phenazine reduction reaction in P. aeruginosa involves multiple enzymes, rather than a specific phenazine reductase, further revealing that phenazine reduction was directly catalyzed by pyruvate and α-ketoglutarate dehydrogenase complexes (Fig. 1c) (18). The involvement of an inner membrane terminal oxidase, *cbb*_3_-type cytochrome oxidase subunit CcoN4, in phenazine reduction in biofilms with tryptone indicates that proton pumping may possibly be coupled with phenazine reduction (Fig. 1c) (19). Given *cbb*_3_-type cytochrome oxidase was not required for phenazine reduction with glucose (20), the energy conversion mechanisms associated with oxygen-terminating EET is modulated in a complex manner. Less ATP generation in oxygen-terminating EET than aerobic respiration is also surmised with thermodynamics in oxygen reduction; because phenazine has a lower redox potential (−0.2 to 0 V versus standard hydrogen electrode, which changes depending on phenazine species) (21) than oxygen (1.23 V versus standard hydrogen electrode), lower energy is acquirable for P. aeruginosa. Another disadvantage of phenazine-mediated oxygen reduction is the limitation of metabolic flux. Because phenazine-based respiration involves an intracellular phenazine reduction reaction, the turnover of which is limited by phenazine concentration, phenazine-based metabolism may be slower than the metabolic flux associated with aerobic respiration when oxygen is abundant. Considered together, aerobic respiration appears to be superior to phenazine-mediated oxygen reduction in terms of both metabolic energetics and kinetics under oxic conditions. Thus, phenazines are thought to act as alternative electron acceptors when aerobic respiratory activity is limited (20, 22).

In contrast, oxygen-terminating EET has an advantage when a spatial oxygen gradient is present. Because phenazine-based respiration consumes oxygen extracellularly, bacteria can sustain their metabolism where oxygen is scarcely diffused into cells but present several tens of micrometers away from the cell (19, 22, 23). Oxygen-terminating EET creates a niche in the anoxic zone that is physically close to oxygen expanding their habitats into niches, thereby challenging the notion that bacterial habitats are physically defined by chemical components inside the cells as assumed in aerobic respiration. Thus, oxygen-terminating EET may sustain bacterial energy conservation in various environments with an oxygen gradient such as biofilms (24, 25), sediments (26), ocean (27), intestine (28), and rhizosphere (29, 30). Another advantage is utilization of oxygen by anaerobic bacteria without aerobic respiratory electron transfer chain. Although strict anaerobes are unable to survive in the presence of oxygen, EET, which enables the utilization of oxygen as an acceptor of electrons from the anoxic zone, assists *F. prausnitzii* to tolerate oxygen in gut mucosa into which oxygen diffuses from epithelial cells, which is advantageous when competing with other anaerobic bacteria (11). The third advantage is that it prevents the generation of reactive oxygen species (ROS) inside cells. Oxygen, which is one of the most abundant electron acceptors existing on Earth, displays the highest redox potential, and thus, utilization of oxygen as an electron acceptor yields higher energy compared with the yield from other electron acceptors. Owing to its high redox potential and high reactivity, oxygen generates ROS that damage various cellular components such as nucleic acids, proteins, and lipids, which presents a major obstacle that bacteria must overcome, causing them to adopt various strategies to avoid ROS damage (31). Thus, oxygen-terminating EET, which facilitates extracellular oxygen reduction, may be an ultimate strategy developed to avoid ROS formation.

### Comparison with fermentation.

Oxygen-terminating EET carries advantages over fermentation in that intracellular redox homeostasis is maintained by oxidation of NAD(P)H while conserving energy. During glucose fermentation in P. aeruginosa, NAD(P)H generated by the Entner-Doudoroff pathway cannot be fully oxidized by subsequent fermentative lactate production; only one NADH is oxidized whereas one NADH and one NADPH are generated by the Entner-Doudoroff pathway (Fig. 1d) (32). Thus, glucose fermentation does not support continuous energy generation in P. aeruginosa, which shows a survival rate of 0.0001% after 7 days of cultivation (17). In contrast, phenazine-based oxygen reduction involves oxidation of NAD(P)H (Fig. 1c), contributing to continuous ATP generation. Similarly, *F. prausnitzii* reduces oxygen with riboflavin and thiols to increase the biomass and ratio of fermentative products with a different NAD^+^/NADH production efficiency (11). This suggests that export of electrons to oxygen facilitates fermentation and energy conservation by balancing intracellular redox homeostasis.

### Comparison with electrode-terminating EET.

Electrode-terminating EET is similar with oxygen-terminating EET in that EET process maintain redox homeostasis while conserving energy. Although electron donation to electrodes is associated with energy conservation, pathways leading to ATP synthesis change depending on the bacterial species: some bacteria synthesize ATP via substrate level phosphorylation, while others synthesize ATP via oxidative phosphorylation using proton motive force. In fermentative bacteria, electrode-terminating EET facilitates fermentation by accelerating NAD^+^ generation turnover as with the oxygen-terminating EET of P. aeruginosa and *F. prausnitzii*. Propionibacterium freudenreichii, Lactococcus lactis, and Enterococcus cecorum change fermentative products to more oxidized products associated with iron reduction in the presence of humic acids (33). *Lactoplantibacillus plantarum*, a lactic acid bacterium carrying a gene locus for EET, which was first reported in Listeria monocytogenes (34), accelerated fermentation turnover by oxidizing NADH to NAD^+^ via electrode-terminating EET, resulting in enhanced substrate-level phosphorylation and biomass production (35). Similarly, in a Gram-negative iron-reducing bacterium, Shewanella oneidensis, gene deletion mutants in F-type ATPase exhibited almost identical growth and current production on electrodes with the wild type, whereas deletion mutants in acetate kinase (*ackA*) and phosphotransacetylase (*pta*), which are required for substrate-level phosphorylation, decreased growth and current production (36). These suggest that substrate-level phosphorylation plays a role in ATP production during EET, which proposed a model that EET process exports protons to cell exterior, resulting in uncoupling with proton motive force (36–38). In contrast, in Geobacter sulfurreducens, proton translocation across the inner membrane, induced by NADH-dehydrogenase and subsequent electron transfer during EET, may lead to the formation of a proton motive force that drives ATP synthesis (39–41). This indicates electron export can couple formation of proton motive force, which is similar with oxygen-terminating EET in P. aeruginosa where proton translocation is possibly coupled with phenazine reduction in CcoN4 (19).

## OXYGEN-TERMINATING EET ACTIVATES P. aeruginosa METABOLISM IN BIOFILMS

A key feature for advantage of oxygen-terminating EET is the presence of an oxygen gradient. As one the most versatile situations regarding oxygen gradients in nature is biofilms (24, 25), here, we introduce recent progress in oxygen-terminating EET in biofilm environments. A biofilm refers to a bacterial community growing on a solid surface, wherein cell-secreted extracellular polymeric substances enable cells to cluster densely. It is estimated that up to 80% of bacterial cells on Earth reside in biofilms (42). Biofilms are formed in a variety of environments associated with biogeochemical cycling processes (43) and infections in plants and animals (including humans) (44); they also have biotechnological applications, including in wastewater treatment and anaerobic digestors (45). Oxygen depletion in the inner sphere of a biofilm, caused by cells in its outer sphere actively consuming oxygen, acts as a critical limiting factor in bacterial metabolism (24, 25, 46–48). Thus, conventionally oxygen distribution has been assumed to define the zone of bacteria to thrive in aerobic biofilm; while aerobic bacteria thrive on the outer surface of biofilms, bacteria in the interior of the biofilm thrive via fermentation or become dormant with lowering metabolic activity (Fig. 1a) (24, 25, 48). In contrast, oxygen-terminating EET leads to a model that cells in the biofilm interior drive their metabolism, even with limited oxygen, by using electron shuttles (e.g., phenazines, riboflavin, and thiols) that accept electrons from cells in the biofilm interior and subsequently diffusing them to oxygen, in the biofilm periphery, which acts as a terminal electron acceptor (Fig. 1a) (19, 22, 23). This model was substantiated via microscopic observation of P. aeruginosa biofilms. Confocal microscopic observation of P. aeruginosa constitutively expressing a stable yellow fluorescent protein (YFP) demonstrated that colony biofilms of phenazine-null (Δ*phz*) mutants showed YFP production within 60 μm from the surface, which corresponds to the oxic zone in biofilm, whereas wild-type producing phenazines expanded the YFP zone to 100 μm from the surface (22). In another study, the incorporation of D7-glucose and D_2_O into colonies was visualized by stimulated Raman scattering microscopic imaging (23). In Δ*phz*, the biofilm interior incorporated deuterium because of pyruvate fermentation without oxygen (32). The wild-type showed more active deuterium incorporation in the biofilm interior, indicating that phenazines had created metabolically active subpopulations showing highly versatile metabolism accompanied by fermentation and phenazine-based respiration in the biofilm interior (23).

## INFLUENCE OF OXYGEN-TERMINATING EET ON BIOFILMS

In biofilms, bacteria employing a variety of metabolic pathways interact with each other, creating a bacterial community that shares roles similar to those of multicellular organisms. Thus, oxygen-terminating EET may contribute to not only the energy metabolism of bacteria, but also the physiology of the biofilm as a whole. Oxygen-terminating EET may enhance the resistance of biofilms to environmental stress, including antibiotic stress. Schiessl et al. revealed that phenazines enhanced ciprofloxacin resistance in P. aeruginosa biofilms (23). Because *cbb*_3_-type cytochrome oxidase, which is involved in phenazine reduction, is required for resistance, phenazine-mediated oxygen reduction in biofilms may contribute to antibiotic resistance. This substantiates the conventional understanding of biofilms, wherein increased phenotypic differentiation due to metabolic diversity may allow parts of the subpopulation to specialize in different tasks, resulting in an overall increase in biofilm resistance against antibiotics (49, 50). Conversely, some antibiotics target and disrupt cell division processes (51); thus, it is also possible that metabolic activation by oxygen-terminating EET increases the antibiotic susceptibility of dormant subpopulations in the biofilm interior. As reported recently (25), because different species show differential responses to environmental conditions, it is essential to directly examine the impact of oxygen-terminating EET on biofilm-based infections, by regulating biofilm based electron transfer. Although methodologies capable of regulating biofilm electron transfers in aerobic biofilms have not yet been developed, electron transfer kinetics in electrode-terminating EET have been regulated via several approaches, such as the addition of redox molecules that act as electron shuttles or cofactors (52, 53), as well as the addition of nanoparticles (54–56), and the use of synthetic biological approaches (57, 58).

Oxygen-terminating EET in biofilms may also influence the metabolism of non-EET-capable bacteria, that are present in biofilms. *Firmicutes* facilitate the production of fermentative metabolites by EET (35) (see Energy Conservation Mechanisms of Oxygen-Terminating EET in Comparison with Other Respiration Methods section). Given that fermentative products can be used as carbon/electron sources by other aerobic bacteria thriving in the biofilm periphery, it becomes evident that enhancement of fermentative products by oxygen-terminating EET may influence the energy and nutrient acquisition of aerobic bacteria in the biofilm. Some *Firmicultes* thrive in the mucus layer of the intestine, forming a biofilm with an oxygen gradient (59). Fermentative products are used by epithelial cells of the host’s intestine (60); thus, oxygen-terminating EET by intestinal biofilms may impact the health of host cells via fermentative products.

## LIMITATION OF OXYGEN-TERMINATING EET IN BIOFILM

Factors that limit the rate and distance of electron transfer in aerobic biofilms remain unclear. Although limiting factors may change depending on conditions, such as bacterial species, bacterial metabolism, oxygen consumption rate, concentration of electron carriers, cell density, and biofilm thickness that surround the biofilm, we summarize possible limiting factors based on progress made in the analysis of biofilm electron transfer in P. aeruginosa.

### Limiting factors involved in electron mediation.

Delivery of shuttling molecules that mediate biofilm electron transfer depends on diffusion process kinetics, which follows Fick’s law, as proposed for oxygen-terminating EET as well as electrode-terminating EET (52, 61). In the case of P. aeruginosa biofilms, the phenazine diffusion model based on Fick’s law reproduces the increase in the metabolically active zone of the wild-type biofilm compared with that of the Δ*phz* biofilm (62). Therefore, parameters determining the redox gradient, which are the concentration of shuttling molecules, diffusion coefficient of shuttling molecules, and reactivity of shuttling molecules with oxygen and bacteria, may act as limiting factors. P. aeruginosa biofilms have adopted various strategies to overcome these limitations. The loss of electron shuttles to the biofilm exterior, which decreases the concentration of shuttling molecules, is a fundamental disadvantage associated with shuttle-based electron transfer. Phenazines produced by P. aeruginosa are retained at concentrations over several tens or hundreds of micromolars, which are sufficient to sustain their metabolism specifically in closed systems (52, 63). A recent study reported that extracellular DNA (eDNA) released by cell lysis can capture phenazines in biofilms that enable efficient biofilm electron transfer even in open systems (64), thereby avoiding phenazine loss and electron transfer limitations. The distribution of phenazines in P. aeruginosa biofilms may also help overcome this limitation. P. aeruginosa produces several phenazines with different local distributions in the biofilms. Phenazines with relatively low redox potentials (phenazine carboxylate [PCA] and phenazine carboxamide [PCN]) are localized in the biofilm interior, while those with relatively high redox potentials (pyocyanin [PYO]) occupy biofilm periphery (65). Considering that reduced PCA and PCN donate electrons to oxidized PYO, depending on its thermodynamics (64), it may be speculated that the redox gradient of PCA/PCN in the biofilm interior and that of PYO in the biofilm periphery are electrically bridged by abiotic electron transfer from PCA/PCN to PYO, resulting in a high electron transfer rate over a long distance, compared with that of the redox gradient formed by a single redox species. In addition, PYO binds strongly with eDNA among phenazines, allowing charge transfer to occur through DNA. This indicates that eDNA may mediate electron delivery from PCA/PCN to eDNA-bound PYO (64).

### pH change in biofilm.

EET accelerates biofilm acidification. When oxygen-terminating EET facilitates fermentation, as confirmed in *Firmicutes*, these acidic fermentation products largely reduce pH (35). In addition to the production of acidic metabolites, secreted protons associated with oxygen-terminating EET may accumulate in the biofilm (36–38). Proton accumulation in the biofilm interior disrupts bacterial metabolism and deactivates bacteria (66, 67), thus, possibly slowing down biofilm electron transfer. Furthermore, pH also influences electron transfer energetics in the biofilm, where a decrease in pH positively shifts the midpoint potential of redox molecules by approximately 59 mV pH^−1^ when the redox species couple one proton to one electron. When the pH at the biofilm interior is reduced because of oxygen-terminating EET, the biofilm component shows a higher midpoint potential than that in the biofilm periphery with higher pH, leading to a thermodynamically unfavorable uphill potential cascade to deliver electrons to biofilm periphery and acts as a potential limiting factor. In addition to these limitations, delivery of the substrate required for bacterial metabolism is also a possible limiting factor in oxygen-terminating EET, whereas diffusion of substrates in the EET-respiration zone, under most nutrient-rich conditions in P. aeruginosa biofilm is sufficiently fast (22).

## SIMILARITY OF OXYGEN-TERMINATING EET WITH ELECTRODE-TERMINATING EET IN BIOFILMS

Creating a niche in aerobic biofilm by oxygen-terminating EET is reminiscent of metabolic activation of EET-capable bacteria distant from electrodes (Fig. 1e and f). EET-capable bacteria located at a distant area from electrode surface in biofilms can drive their metabolism without direct contact with electrode, transferring electrons to electrodes mediated by biofilm components (Fig. 1e). Given its significance, it will be of great interest to examine oxygen-terminating EET in other bacteria besides P. aeruginosa and *F. prausnitzii*. For example, it would be of interest to test a human pathogen, Listeria monocytogenes, because it is suggested that L. monocytogenes utilizes EET pathway in oxygen reduction when the genes for terminal oxygen reductases in aerobic respiration are deleted (68). S. oneidensis MR-1 conducts electrode-terminating EET using outer membrane *c*-type cytochrome complexes (69–71). Although oxygen-terminating EET has not been observed in this strain, the reaction centers in outer-membrane *c*-type cytochrome complexes are hemes, which can be abiotically oxidized by oxygen (72, 73), allowing aggregations of S. oneidensis MR-1 to transfer electrons on a micrometer scale via these cytochromes that are associated with cell–cell physical interactions (74, 75). Teal et al. developed aerobic biofilms and demonstrated that expression of *mtrB* was higher in its oxygen-limited interior compared to that in its periphery using reporter strain via confocal microscopy (76), suggesting that S. oneidensis MR-1 survives and synthesizes proteins in the biofilm interior with scarce oxygen. Because MtrB is a β-barrel protein holding cytochromes to deliver electrons across outer-membrane as a part of EET pathway (77), the high expression of *mtrB* in the biofilm interior implies that electron transfer occurs in the biofilm interior.

In addition, a microbiome in oral plaque was proposed to mediate EET termination with oxygen as the electron acceptor (78). Although neither electron conduction nor oxygen-terminating EET has been demonstrated in oral plaque, some bacteria in plaque have cell-surface redox regents showing electrode-terminating EET capability (79, 80). Bacteria in the biofilm interior exhibited a lower redox potential than those in the biofilm periphery, showing that a thermodynamically favorable cascade connects an anoxic biofilm interior to the oxic periphery (78, 81).

## CONCLUSION

This review reflects our attempt to compile research findings detailing recent progress made in the understanding of oxygen-terminating EET and its links with energy metabolism, primarily based on a model biofilm of P. aeruginosa. Oxygen-terminating EET confers several advantages over aerobic respiration and fermentation by allowing bacteria to create a niche in biofilm-based remodeling of metabolic activities. Although bacterial habitat is conventionally assumed to be defined by chemical components incorporated into cells, long-range electron transfer enables bacteria to thrive where oxygen is absent intracellularly but present near the cell, expanding their habitat. This notion is analogous to unique cable bacteria that electrically couple sulfide oxidation with oxygen reduction via multicellular filaments on a centimeter scale (82). Although this type of respiratory coupling has been limited to unique cable bacteria, the idea of oxygen-terminating EET opens up the possibility that EET-capable bacteria may proceed similarly in various environments in the presence of an oxygen gradient such as sediments (26), ocean (27), intestine (28), and rhizosphere (29, 30). We hope that this review will provide a basis that links bacterial physiology with electron transfer in the presence of chemical gradients formed by soluble terminal electron acceptors.
